# Georgia State Medical Association

**Published:** 1894-05

**Authors:** 


### SOCIETY REPORTS.

### GEORGIA STATE MEDICAL ASSOCIATION.

ATLANTA, GA., April 18, 19, 20, 1894.

The Association convened in Concordia Hall, and was called to order by the President, DR. W. H. ELLIOTT, of Savannah, at 10 A. M.

Prayer was offered by the Rev. I. S. Hopkins.

An address of welcome was delivered by the Hon. N. J. Hammond, of Atlanta, and President Elliott responded thereto in behalf of the Association.

DR. J. B. S. HOLMES, of Atlanta, took the chair, and President Elliott delivered his address. He selected for his subject "The Duty of the Physician to His Patient." (See page 129.)

The reading of papers was then proceeded with, and the first paper read was by DR. W. C. HUMPHRIES, of Acworth, entitled "The Treatment of Pneumonia," in which he confined himself mostly to the acute lobar variety. The majority of cases with which he comes in contact assume from the beginning the sthenic type and require active or depleting measures. If called to treat the patient in the first stage of the disease, he resorts to moderately active catharsis and an agent that will arouse the dormant accretions and assist nature to eliminate from the system the effete products produced by the disease. For this purpose he finds no better remedy that calomel, and usually prescribes from six to ten grains to an adult in combination with Dover's powders or some preparation of opium and bismuth subnitrate. His next effort is directed to reducing the force and frequency of the heart's action, and for this purpose he has tried several remedies, such as aconite, gelsemium, the coal tar derivatives, etc., but none of them have given so great satisfaction or such uniform results in his hands as veratrum viride. He uses this oftener than any other heart sedative in this disease and it seldom ever fails him. He does not give it to such an extent as to produce nausea, but in small and often repeated doses. With veratrum he combines turpentine in the form of an emulsion. The author then dwelt upon external applications in the treatment of the disease.

DR. O. H. BUFORD, of Cartersville, followed with a paper on the "Fluid Extract of Jaborandi as an Abortive Treatment in Pneumonia." The author thinks that in this drug we have a remedy that in many cases wiil force a crisis when given early in the congestive stage. He had used it for several years with good effect. After giving fluid extract of jaborandi, he follows it with quinine and stimulants if indicated. The author then reported three cases in which he used this treatment.

DR. T. M. GREENWOOD, of Mineral Bluff, read a paper on the "Abortive Treatment of Pneumonia." He said his mode of treating the disease was to apply a mustard plaster over the entire portion of the lung affected. He usually makes or has them made under his special directions, using the purest brand mixed with water only, letting the plaster remain on the patient for six hours. He next investigates the condition of the bowels, the secretions in general, and, if necessary, gives a mercurial. If this does not cause a free evacuation of the bowels, he administers Epsom salts or castor oil. He next prepares a solution of salycilate of soda, containing about sixty grains of the soda to an ounce of water, giving one teaspoonful every two or three hours, according to the intensity of the pain and the amount of fever. Expectorants in his hands have done little or no good, generally doing harm, the great objection to this treatment being, when long continued, its rejection by the stomach. He considered this treatment abortive, for in his hands it has either been abortive or curative. A trial of over fifty cases has given varying success without a single death.

DR. C. S. WEBB, of Atlanta, called attention to the fluid extract of ergot to reduce temperature in the first stage of pneumonia. Ergot is to some extent a heart depressant. It slows the heart and reduces the temperature, and in his opinion is not so dangerous a drug as the fluid extract of jaborandi.

DR. R. R. KIME, of Atlanta, said it was absolutely essential in the treatment of pneumonia to distinguish between the first stage of the affection and that of absorption of the circulation after consolidation had taken place, and adapt the treatment to these conditions, otherwise failures would be made. Bleeding and the use of coal tar derivatives should have a limited place in the treatment of the disease. It was worse than folly to tax the heart to its utmost capacity by the use of cardiac remedies. But the physician should increase the power of the heart, equalize the circulation in order to get rid of the material in the lung. At this stage many cases could be tided over the critical period by the hypodermatic use of strychnine.

DR. W. A. DOUGHTY, of Augusta, formulated the treatment of the disease under four heads: (1) Give the patient plenty of fresh air; (2) keep him clean; (3) feed him; and (4) come just as near as possible to letting him alone.

### AFTERNOON SESSION.

DR. H. WILLIAMS, of Macon, read a paper entitled "A Case of Sarcoma of the Ilium following a Railway Injury." The patient was thirty-two years of age, strong and healthy, a trainmaster by occupation. There was a wreck on his division, and it was important that the roadway should be rapidly cleared, and so he went to the scene of the accident. A guide rope was attached by one end to a locomotive and by the other to some part of the wreck, so that the power of the locomotive could be used in removing the obstacle. The patient was standing by this rope, it broke under the strain, he became entangled and was dragged some distance by the engine, striking his right buttock against the corner of a cross-tie. He felt no inconvenience from the accident except a momentary pain following a contusion near the exit of the great sciatic nerve, and returned to work. A month later he consulted Dr. Williams for a pain in his right buttock. One day while using deep injections the author thought he felt the end of his needle scraping denuded bone. This he mentioned to the patient and then received the first intimation of the injury received at the wreck. From this he constructed a theory that the bone had been injured, and denuded of periosteum was pressing upon the sciatic nerve at the notch in the pelvis. The doctor suggested the possibility of an operation, which at this time was declined. About six weeks or two months later he operated. Beginning his incision as for aneurism of the sciatic artery, at the posterior superior spine of the ilium, it was carried over the top of the larger tumor of the greater trochanter, and the tissues were dissected down to the tumor. It was situated on the flange of the ilium just above the exit of the sciatic nerve and attached to the bone, though unattached to the muscles over it. The upper and posterior third of the great sciatic notch was denuded of periosteum and a stellate fracture radiating from the notch upward and backward for an inch and one-half was found, the bone being pressed slightly inward upon the sciatic nerve. In attempting to cut out the tumor a cavity in it was opened followed by a gush of fluid and a small clot. He feared he had opened an aneurismal sac. Running his finger into this sac to find the opening of the artery, he found the interior of the cavity encrusted with small spicula of bone. There was no collapsing of the cavity nor any further escape of fluid after the first gush from it. He removed several pieces of denuded bone and as much as possible of the tumor. Not a drop of pus was found in the growth. The operation was a very bloody one and the patient was very much depressed. The wound was packed with iodoform gauze and dressed antiseptically, the patient put between blankets, stimulants administered hypodermatically and hot bottles used to bring on reaction. Examination of the growth disclosed it to be a subperiosteal sarcoma of the mixed type. The patient did well for weeks following the operation, but died some forty-three days after the operation, and nearly eight months after the accident.

DR. J. MCFADDEN GASTON, of Atlanta, read a paper entitled "Operation for Fibro-Cystic Sarcoma, Involving the Right Inferior Maxillary Bone." (See page 142.)

DR. ARTHUR G. HOBBS, of Atlanta, read a paper entitled "Treatment of Pterygium with the Galvano-Cautery." (To be published in this JOURNAL.)

DR. M. B. HUTCHINS, of Atlanta, read a paper entitled "Malignant Growths of the Skin: Their Diagnosis and Treatment." (To be published in this JOURNAL.)

DR. J. W. HALLUM, of Carrollton, contributed a paper on "Chronic Sore Legs and How to Cure Them," in which he said that chronic sore legs were the results of a disease that we often meet in our healing journey. In the remarks of his paper he excluded specific and malignant ulcers, and discussed a simply chronic ulcer that has refused to heal from all efforts of nature that have been brought to bear upon it. The author had been curing such ulcers by painting them with carbonate of lead and linseed oil, in the following proportion:

| | |
|---|---|
| Pure white lead (ground in oil)...................................... | ℥ xv. |
| Raw linseed oil.................................................... ..... | ℥ vi. |

Mix well, and sign, paint the ulcer once or twice a day after washing it with warm water. Dry well before painting. The best thing to apply the remedy with is a camel's hair brush He was not able to tell how this application effects a cure; but carbonate of lead is sedative, astringent, and probably possesses disinfectant powers, all of which the author considers quite essential in the cure of these ulcers. The shortest time he has been able to accomplish a cure by this method was six days. The ulcer was two and one-half inches in diameter and of three years standing, but had not penetrated the entire true skin.

SECOND DAY—MORNING SESSION.

DR. H. PERDUE, of Barnesville, read a paper on "Diagnosis." He said the ability to make an early and quick diagnosis should be sought, and, if possible, attained by every physician. It enables one oftentimes to abort disease, and even when this cannot be done the violence of the attack may often be modified and its duration abridged. Thoroughness should be the aim of every physician. None should be satisfied with attainments short of the ability to make a diagnosis through scientific and practical methods of research.

DR. LUTHER B. GRANDY, of Atlanta, read a paper entitled "Suture of the Tendo Achillis," in which he reported a case and exhibited the patient.

Patient, age twenty-five, severed tendo Achillis with adze. United ends of tendon with silk and catgut ligatures. Dressed in plaster Paris, which was removed at end of four weeks. In a few days patient received fall and tendo was disunited.. Again Sutured, using silk worm gut. Ankle put up in plaster again and allowed to remain six weeks. Ultimate recovery excellent, with no impairment of function.

Dr. J. B. S. Holmes then read a paper entitled "Making and Closing the Incision in Abdominal Surgery." (To be published in this Journal.)

Dr. F. W. McRae, of Atlanta, contributed a paper on "Appendicitis with Report of Cases." (To be published in this Journal.)

Dr. Samuel C. Benedict, of Athens, delivered the orator's address. He selected for his subject "Suggestion and Its Therapeutic Uses." By suggestion is meant simply an impression made from without, through one of the five senses, upon the cells of the cerebral cortex; it is a sensory impression by which either motor phenomena only are developed, or mental action, such as revival of memory. In some instances a language used in childhood and long since forgotten has been brought again into speech as clearly as though never lost. Auto-suggestion arises without external impressions and is typical in dreams of delirium. Now, as explantory of such phenomena, a working hypothesis has been formulated by Hudson which explains better than any previous theory the causes for such phenomena. His reasoning is that man has two minds, one of which he calls the objective and one the subjective mind, and, further, that the subjective mind, which is the storehouse of the memory and the seat of the emotions, is constantly amenable to control by suggestion, and is incapable of inductive reasoning.

Dr. P. R. Cortelyou, of Marietta, reported an interesting case of inflammation of external auditory canal following facial erysipelas in a woman sixty-eight years of age.

Dr. Dunbar Roy, of Atlanta, read a paper on "Chronic Otorrhea, Its Significance and Treatment." (To be published in this Journal.)

Dr. J. H. Shorter, of Macon, read a paper entitled "Treatment of Cataract." He said all operations for removal of cataract have been by one of three methods: Depression, discission or extraction. These three methods were then described in detail and the indications for each method pointed out.

DR. H. MCHATTON, of Macon, followed with a paper on "Teething." No one denies that dentition is a physiological process, *pari passu.* As the infant departs from its natural state, teething is increased in violence. The infant who is fortunate enough to have a mother who is physiologically perfect, and who treats her child as nature intended, does not teeth the first year, but is liable to the second summer. The one who has to be partially bottle-fed teeths oftener and earlier than the above mentioned. The one that is bottle-fed from the start, and exclusively, teeths, as a rule, early, and in many cases fatally. The author knew that most practitioners would agree that dietetic errors are the cause of nearly all digestive disorders in the infant, and that these disorders are in the beginning and non-inflammatory stage most amenable to appropriate treatment, but by neglect they become the worst type of diseases that we have to contend with. The author then quoted from standard authors in regard to the subject of teething.

DR. RICHARD DOUGLAS, of Nashville, addressed the Association by invitation, on "Surgical Shock."

DR. R. H. TAYLOR, of Griffin, read a paper on "An Operation for Hemorrhoids." He describes the operation as follows: After preparing the patient for operation in the usual way, fix the hemorrhoid with vulsellum forceps or a stout tenaculum, which is firmly held by an assistant. Instead of cutting a groove around the base of the pile for your ligature in the usual way, make an elliptical incision through the mucous membrane, including the hemorrhoid in the ellipsis. Turn back a small flap of mucous membrane from the base of the tumor. Now, have the assistant make firm traction on the pile while you pass a double ligature of stout chromicized catgut through the pedicle, which may be much reduced in size in some instances by separating it from the muscular coat of the bowel from below upward. Firmly secure the ligature and cut away the tumor as close to the ligature as possible, only leaving enough stump to safely prevent slipping of the ligature. Then close the flaps over the ligated stump with a suture of the chromicized catgut. This leaves you with a closed wound which, if aseptic, will practically heal in four days.

DR. R. P. COX, of Rome, read a paper entitled "Sacrificial

Surgery of the Ovaries, Tubes and Uterus for Uterine Fibroids and Uterine Cancer." In the treatment of uterine fibroids uterine cancer, radical interference is often the best hope for the one and always the only hope for the other. The rather indefinite, though usually given, moral ground of justification for excision of the pelvic organs is to save life, to preserve from destruction a patient's usefulness or her capacity for the rational pursuit of happiness, or to relieve serious and permanent impairment of these natural rights. The author said that any medical man who advises or performs these radical operations on vague indication, without the ability to give an honest and reasonably intelligent opinion as to the necessity and probable results, and, in doubtful cases, without the sanction of skillful and conscientious counsel, has failed in his duty and dishonored his profession. The author concludes that in nearly all cases supposed to be operable carcinoma uteri, we should try to exclude cancer by the microscope, but if this examination confirms the diagnosis of cancer, we should proceed at once to amputation, or in a certain sense, excision, by the galvano cautery, and not by vaginal hysterectomy. Abdominal hysterectomy is indicated only in certain cases where the malignant disease is complicated by enlargement, such as by myoma or pregnancy.

DR. W. B. GILMER, of Macon, read a paper entitled "Drainage of the Peritoneal Cavity with the Use of the Siphon Pump."

THIRD DAY—MORNING SESSION.

DR. R. M. HARBIN, of Calhoun, read a paper entitled "Trephining in Head Injury with Paralysis of Opposite Arm, Followed by Fungous Cerebri," and reported an interesting case which he considered a distinct indication for operative interference. The patient was twenty-five years of age, and in a fight was struck upon the head with the heavy end of a thirty ounce billiard cue, and was carried home in an unconscious condition in about two hours thereafter, having vomited a number of times. The patient was seen on the third day after the injury with the left arm completely paralyzed, sensation being normal. A diagnosis of compression of the brain was made, probably due to a blood clot. Trephining was done on the seventh day after the injury.

Dr. Harbin drew the following deductions from the case:

1. That this patient would almost certainly have died in the first instance had not the clot been removed.

2. That there was a positive indication for trephining.

3. That it furnished a striking example of the practical value of the applied rules of topographical anatomy of the brain.

4. That the prevention of fungous cerebri is more important than the cure, and is not to be looked for without proper precaution.

5. That the occurrence of fungous cerebri is very unusual in such a small trephine wound without injury to the dura mater.

DR. GEORGE H. NOBLE, of Atlanta, contributed a paper on "Phlegmasia Alba Dolens," and DR. J. M. HULL, of Augusta, one entitled "Foreign Bodies in the Larynx."

AFTERNOON SESSION.

DR. WALTER A. CROW, of Atlanta, read a paper entitled "Cancer of the Uterus, the Remote Result of Operative Interference," in which he reported a series of cases that had come under his care recently, and which served to impress two facts: (1) The common occurrence of this disease, especially in women approaching the menopause, and (2) the great disposition to overlook their true nature or probable tendency, and to incline to use palliative measures until the disease assumes a malignant form, and we thereby lose our golden opportunity of effecting a cure in most cases.

DR. H. W. STAFFORD, of New York, read a paper, by invitation, on "The Extraction of Clear Lenses for Myopia," and reported five interesting cases. Previous to describing the operations, he related the circumstances which led him to believe that the extraction of the lense, whether clear or not, would arrest the progress of myopia.

"Responsibility and Relation of the General Practitioner to Gynecology, Preventive and Non-Preventive." This was the title of a paper read by DR. R. R. KIME, of Atlanta, in which he said to properly prepare for special work in gynecology, requires years of practical experience in general medicine and obstetrics. The most successful practitioner is one who seeks to know and eliminate as far as possible the cause of disease. In gynecology work it is not so often a question of eliminating the primal cause of disease as it is to correct the results of antecedent causes. To illustrate: Pyosalpinx is not a primal cause, but the result of a previous inflammation, simple, infectious or specific. Pelvic peritonitis in the female is not idiopathic, but the result of diseased conditions elsewhere.

Having cited some of the many instances in which the general practitioner may prevent disease and injury to the female and lessen gynecological work, the author then pointed out a few instances in which his duty is reversed, and for the sake of brevity presented them in the form of *don'ts*.

DR. J. G. EARNEST, of Atlanta, reported a case of extra-uterine pregnancy in a woman thirty-two years of age, the mother of three children.

DR. J. C. AVARY, of Atlanta, read a paper entitled the "Tampon in Gynecology," and DR. BERNARD WOLFF, of Atlanta, one entitled "A Plea for the Closer Recognition of Dermatology as a Specialty."

DR. J. MCFADDEN GASTON, of Atlanta, introduced the following resolution, which was unanimously adopted:

WHEREAS, The American Medical Association at its last meeting provided for an expression in regard to the Code of Ethics from the different State Associations, to be laid before the forthcoming meeting at San Francisco, it is hereby

*Resolved*, That the Medical Association of Georgia reasserts its adoption of, and conformity to, the Code of Ethics of the American Medical Association, as heretofore recognized by that body, and authorizes this record of its adherence to the same.

Next year the Association will meet in Savannah on the third Wednesday in April.

